# Chronic Submandibular Sialadenitis: A Case Report

**DOI:** 10.7759/cureus.114945

**Published:** 2026-08-21

**Authors:** Ramachandra Reddy Gowda Venkatesha, Vinitha Ganesh, Karthik Rajaram Mohan, Saramma Mathew Fenn, Sindhuja Rajalingam

**Affiliations:** 1 Oral Medicine and Radiology, Vinayaka Mission's Sankarachariyar Dental College, Vinayaka Mission's Research Foundation (Deemed to be University), Salem, IND

**Keywords:** salivary calculi, salivary gland calculi, salivary gland stone, submandibular glands, submandibular sialadenitis

## Abstract

Sialoliths are mineralized deposits of calcium salts, primarily calcium in the form of hydroxyapatite, a smaller percentage of potassium, magnesium whitlockite, brushite, and ammonium. Sialoliths result in sialadenitis when they obstruct salivary flow within the lumen of the duct, resulting in inflammation of the salivary gland. This article highlights the clinical signs and symptoms of submandibular sialadenitis caused by a sialolith in a 22-year-old male and the importance of a non-invasive retrieval method for submandibular sialolith. The technique involves milking or directly massaging the submandibular salivary gland by bimanual palpation, effectively preventing invasive surgical procedures of submandibular gland excision.

## Introduction

Sialolithiasis is the process of formation of salivary calculi within the major salivary glands. A sialolith is twice as likely to affect males than females [1]. More than 80% of sialolithiasis occurs in the submandibular gland, followed by the parotid gland at 6% and the sublingual and other minor salivary glands at 2%. The rarity of sialolithiasis in children, with a prevalence of only around 3%, underscores the unique nature of this condition [1]. Most cases of sialoliths are reported in adults, with the condition rarely occurring in the pediatric age group [1]. Most salivary sialoliths are unilateral, and multiple calculi in the submandibular gland are rare [1]. Prolonged blockage of the duct by a sialolith can cause damage to the gland, resulting in a permanent reduction of salivary function [1]. Forty percent of the parotid and 20% of submandibular calculi are radiolucent; diagnostic sialography is necessary to diagnose a sialolith in such cases [1]. A sialolith generally has a round to oval form, a rough surface, and a whitish-yellow color [1]. Sialoliths are composed of calcium salts, primarily calcium phosphate, with carbonates in the form of hydroxyapatite in smaller amounts and a smaller percentage of potassium, magnesium, and ammonium salts [2]. Submandibular stones are 18% organic and 82% inorganic. The core of a sialolith does not contain any bacterial component; the organic material typically consists of various amino acids and carbohydrates [2]. Aoun and Maksoud reported an oblong-shaped submandibular sialolith resembling the shape of a canine tooth [3]. Badash et al. stated the use of transoral robotic surgery (TORS) and the DaVinci system for surgical retrieval of submandibular sialolith with the benefit of the highly precise surgical incision and better preservation of anatomical structures [4].

## Case presentation

**Figure 1:**
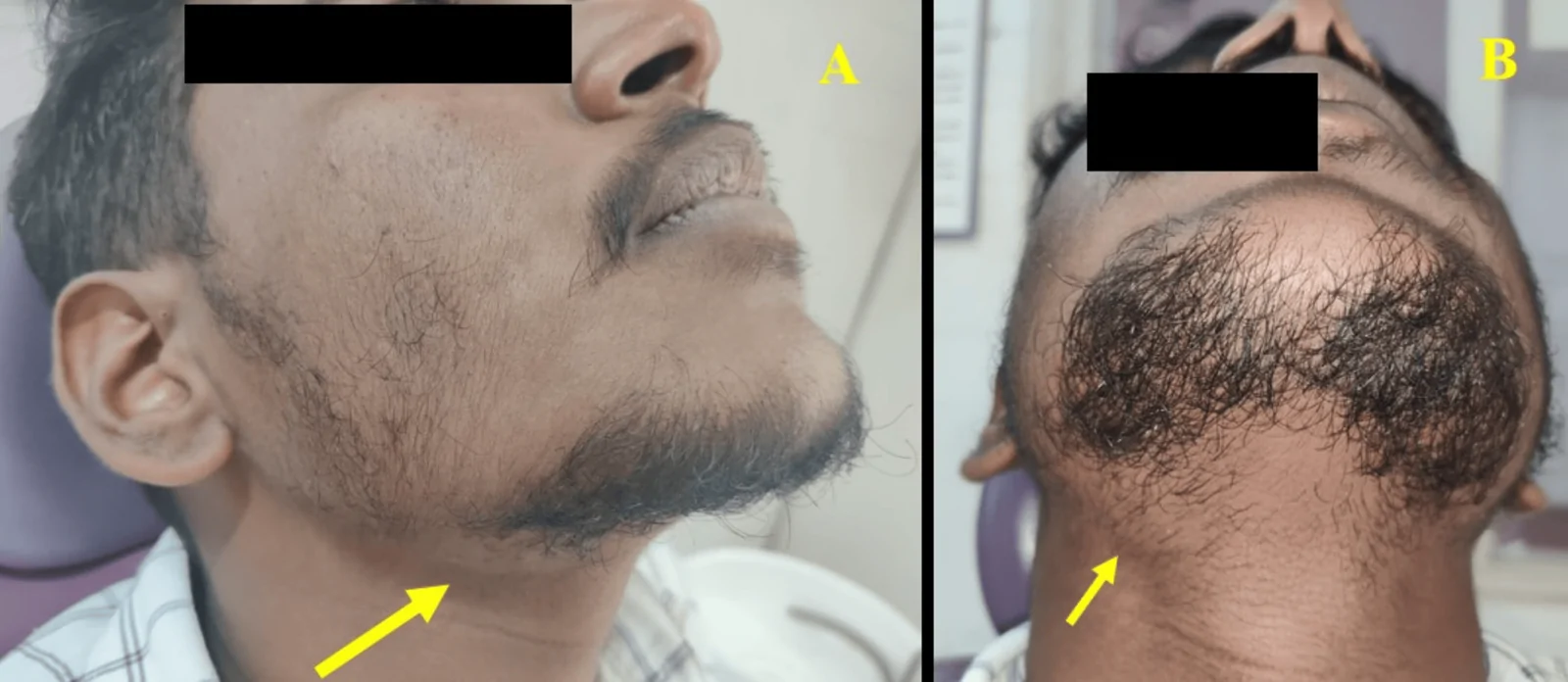
A. Extraoral right lateral profile view revealed an extraoral swelling measuring approximately 1.5 cm x 1 cm involving the right submandibular gland region beneath the lower border of the angle of the mandible (yellow arrow); B) Extraoral submentovertex view revealed a swelling measuring about 1.5 cm x 1 cm in the right lateral aspect of the neck in the region of the right submandibular gland (yellow arrow).

A 22-year-old male reported a chief complaint of pain and swelling in the right lower side of his neck and under the surface of his tongue three days prior to presentation (Figures 1A, 1B).

**Figure 2:**
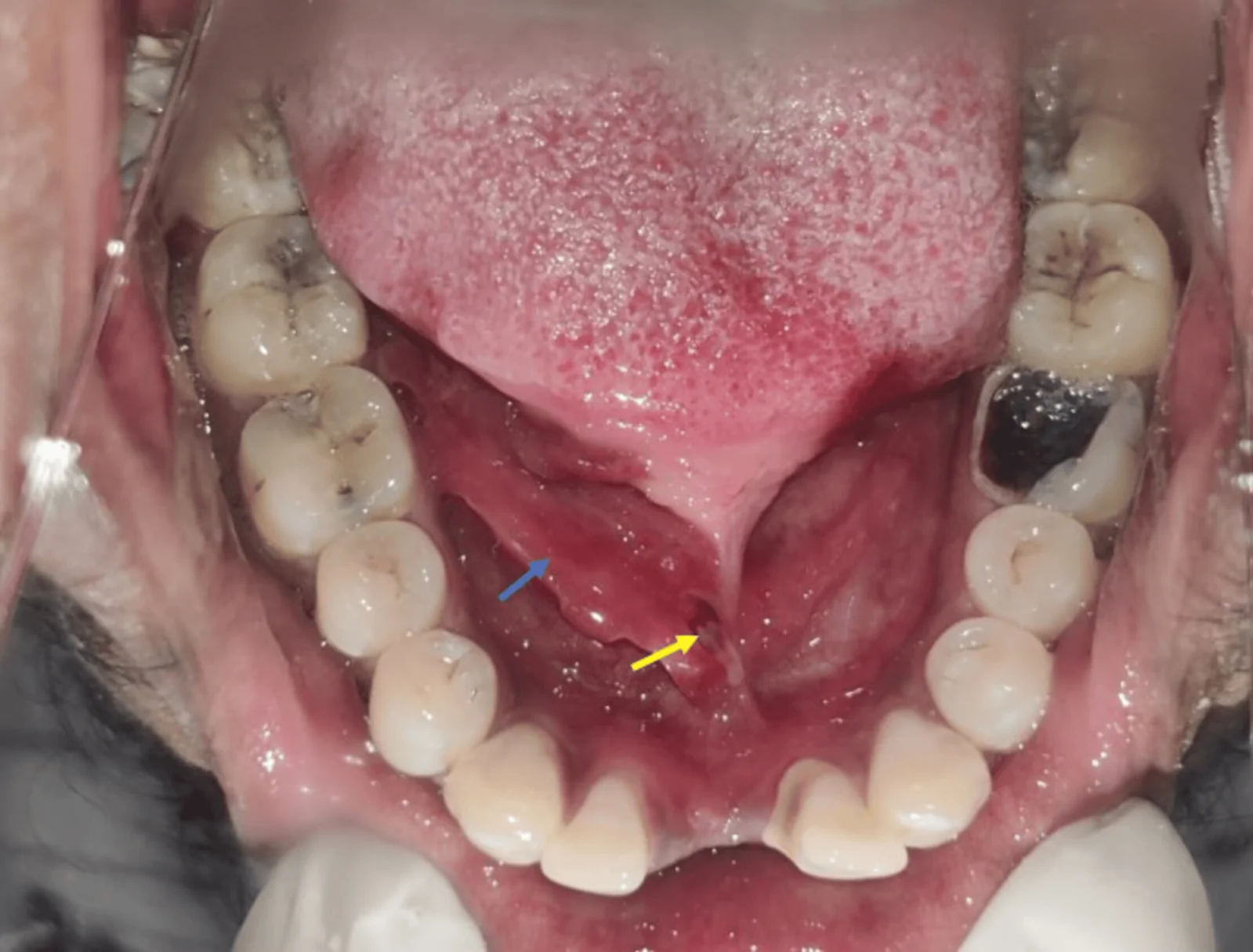
Intraoral clinical examination revealed a flabby tissue extending posteriorly from the mandibular second molar anteriorly till the lingual aspect of the edentulous right mandibular central incisor region (blue arrow) and a salivary oro-fistula in relation to the right lateral aspect of the mandibular labial frenum (yellow arrow).

The pain, which was acute in onset, pricking, and non-radiating, significantly hindered his ability to eat. The patient had no history of other associated comorbidities, such as diabetes or hypertension. The patient did not give any history of harmful oral habits such as smoking or chewing tobacco or consumption of alcohol. The patient provided no history of any drug allergy. Extraoral examination revealed extraoral swelling in the right submandibular region. The right submandibular lymph node was 2 cm in diameter and tender, mobile, and firm in consistency. Intraoral examination revealed inflamed flabby tissue concerning the floor of the mouth in the 43 to 47 regions and a salivary oro-fistula about the right lateral aspect of the mandibular labial frenum, near the sublingual caruncle region, into which the orifice of the submandibular salivary gland duct opens (Figure 2).

**Figure 3:**
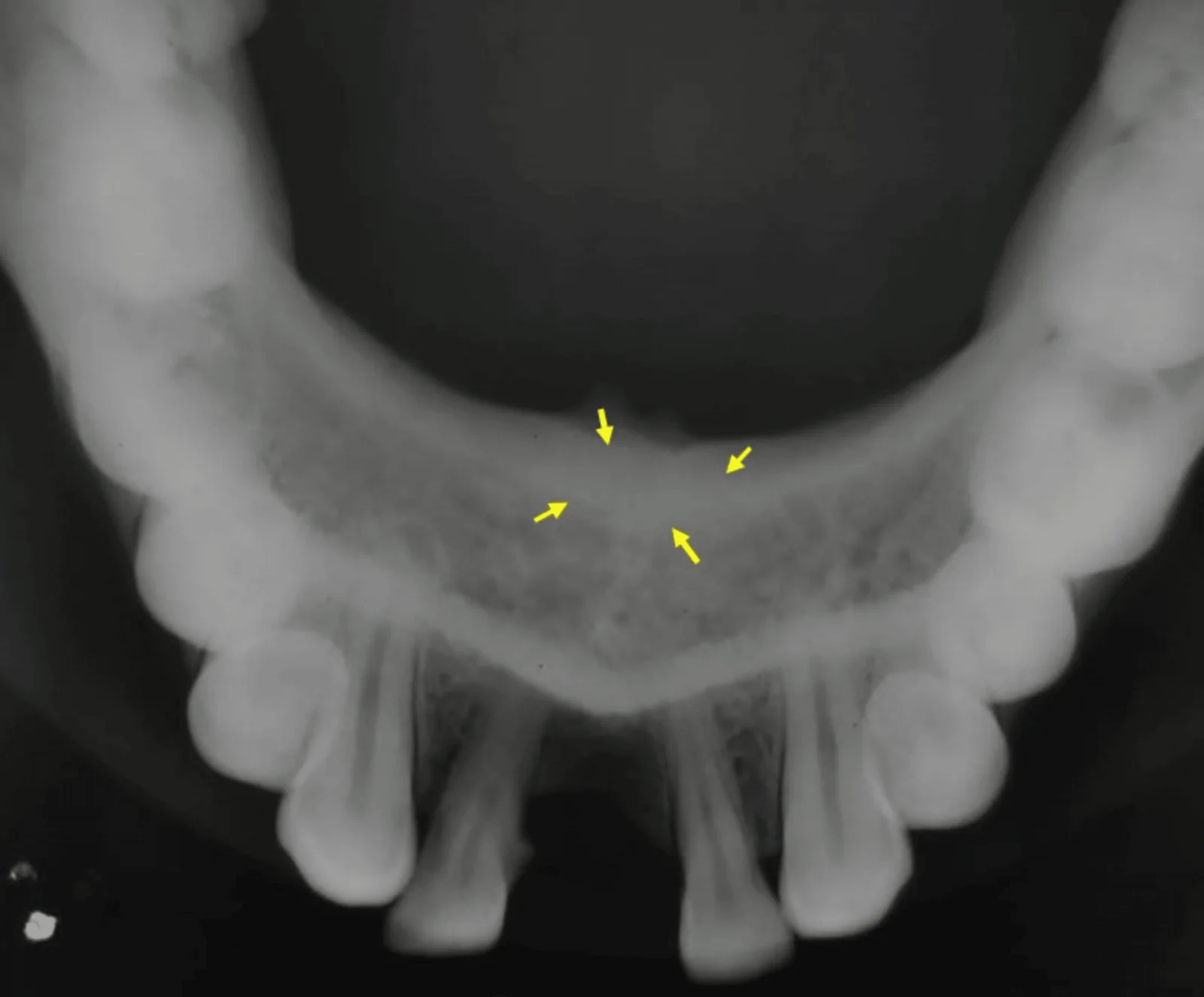
Topographic mandibular occlusal radiograph revealed a dense radiopaque mass superimposing the area of genial tubercle (yellow arrow).

Based on the patient's chief complaint, history, and extraoral and intraoral clinical findings, a provisional diagnosis of an enlarged submandibular lymph node due to submandibular sialadenitis was arrived at. The topographic mandibular occlusal radiograph revealed a radiopaque area projected near the mandible's lingual border concerning the right mandibular canine region (Figure 3).

**Figure 4:**
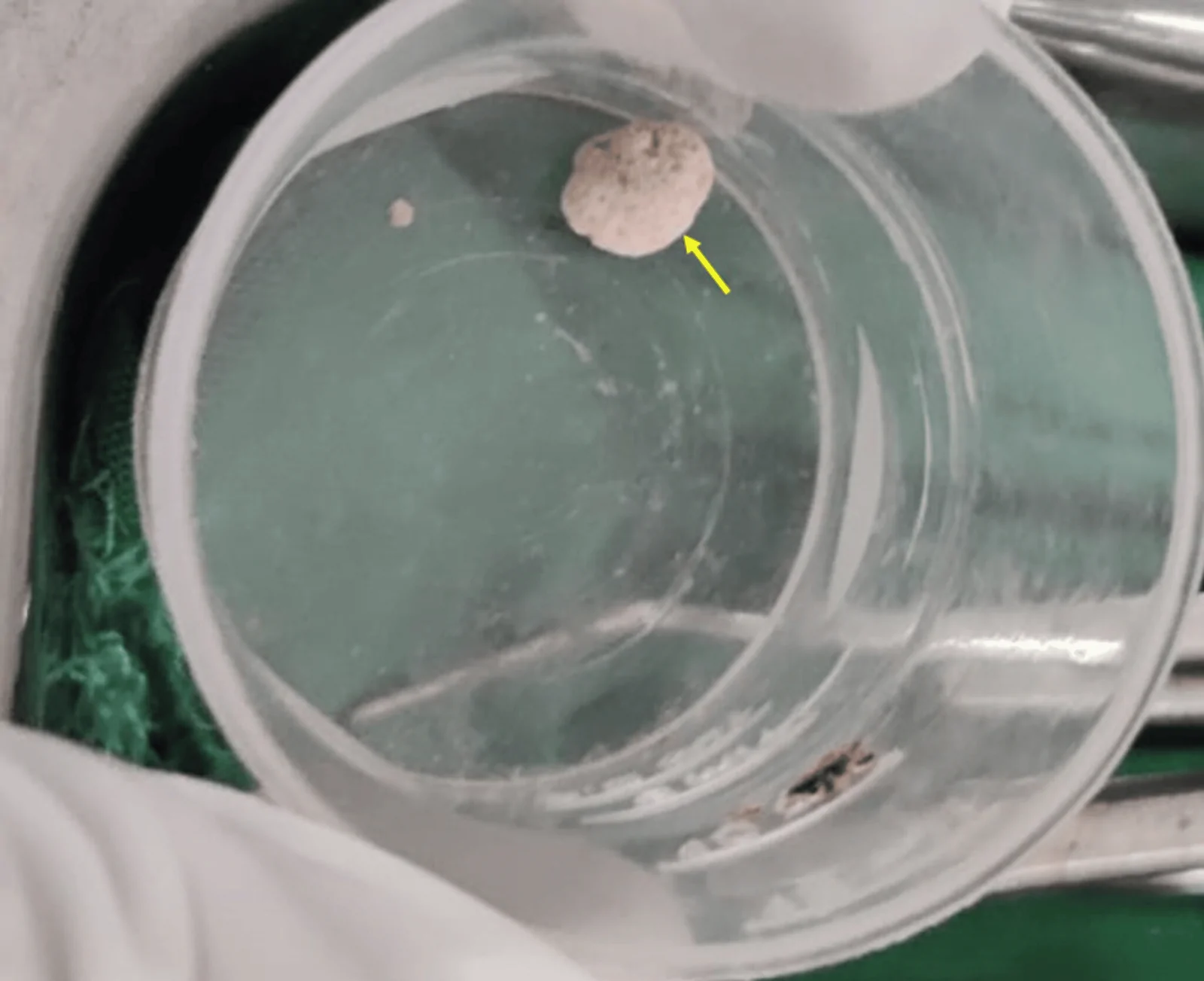
A submandibular sialolith was manually retrieved by gentle massaging of the right submandibular salivary gland (yellow arrow).

The orifice of the submandibular gland duct appeared inflamed and showed discharge on palpation. Based on the history, clinical, and radiographic features, the final diagnosis was made as chronic submandibular sialadenitis secondary to a submandibular sialolith. The differential diagnoses for radiopaque mass include calcified submandibular lymph node and phlebolith. Phleboliths usually occur multiple and contralateral. A submandibular sialolith usually appears unilateral, rarely bilateral. The radiographic outline of the submandibular lymph node is generally smooth and regular but rough and irregular in the case of a submandibular sialolith. The right submandibular gland is massaged gently by bimanual palpation. Bimanual palpation of the right submandibular gland is achieved by placing the forefinger on the oral mucosa involving the floor of the mouth, extending from the lingual surface concerning the right mandibular second molar, migrating gradually anteriorly along the floor of the mouth till the right mandibular incisor region, and simultaneously placing the forefinger of the other hand on the external aspect of the right lateral surface of the neck beneath the lower border of the mandible involving the right submandibular salivary gland. A submandibular sialolith, which measured about 5.5 mm x 6 mm, was gently retrieved (Figure 4).

After manual retrieval of the sialolith, the following analgesics and antibiotics were prescribed: tablet (tab) amoxicillin (500 mg) with potassium clavulanate (125 mg) twice daily every 12 hours for one week; tab metronidazole 400 mg thrice daily every eight hours for one week; tab aceclofenac 100 mg twice daily every 12 hours for five days; and tab pantoprazole 40 mg once daily for a week. After a week of follow-up with analgesics and antibiotics, the patient was relieved of his discomfort due to pain and swelling.

## Discussion

Sialadenitis refers to the inflammation of the salivary gland. Chronic sialadenitis involves persistent or recurrent inflammation of the salivary glands, often due to obstruction of salivary flow caused by salivary stones or strictures, and usually clinically presents with swelling of the affected salivary gland [1]. Salivary gland lithiasis (sialolithiasis) is a condition where calcified concretions occur within the salivary glands, usually the submandibular gland. Submandibular stones occur generally in Wharton's duct and are 8-10 mm in diameter [2]. Giant-sized sialoliths are more than 1.5 cm in diameter [3]. Sialoliths comprise a central organic layer of glycoproteins, mucopolysaccharides, and cellular debris and a peripheral inorganic layer comprising calcium carbonates and calcium phosphates, magnesium, iron, manganese, and copper [4]. Sialolithiasis is the formation of salivary stones within the major salivary gland ducts, namely submandibular (Wharton's), sublingual (rivinus), and parotid (Stenson) ducts.

Sialendoscopy combined with a minor surgical extraction of submandibular and parotid sialoliths is safe and productive. About 80%-95% of sialoliths occur in submandibular salivary glands [5]. Sialoliths have an ample unstructured amorphous material called "aspidinic" hydroxyapatite layers with little brushite [5]. Grases et al. stated that phytate, an anticrystallization chemical, plays a vital role in sialolith formation [5].

The longer, tortuous, and larger caliber of the submandibular duct, the slower or anti-gravity flow rate of saliva due to the anatomical location of the submandibular salivary gland and its angulation around the mylohyoid muscle, and the higher alkalinity, mucin, and calcium content of saliva are inherent factors contributing to the formation of a submandibular sialolith [6]. Reddy R reported a case of submandibular steinstrasse, which refers to multiple stacked stone fragments in a column within the Wharton duct causing complete blockage of the submandibular duct, visualized in high-resolution ultrasonography [7]. The process of formation of salivary stones (sialolithiasis) is the most common cause of chronic submandibular sialadenitis [8]. Small stones can be removed manually by bimanual palpation [9]. Submandibular stones usually form around a central nidus of mucous frequently [9]. A retrograde theory states that any substance or bacteria of the oral cavity that has migrated into the salivary ducts can act as a nidus for further calcification, resulting in sialolith formation [9]. The scanning electron microscopic analysis of sialolith revealed plate- or needle-like hydroxyapatite, calcium carbonate apatite, calcium oxalate dihydrate, tricalcium phosphate crystals, and rhombohedral-shaped whitlockite [10].

The incidence of sialolithiasis in the Chinese population is 0.45%-1.20% [11]. About 80%-90% of salivary gland stones occur in the submandibular gland, and the submandibular gland stones in the anterior middle segment of the duct are retrieved through an intraoral incision or stone basket with endoscopic assistance [11]. The management of giant-sized submandibular sialoliths involves sialadenectomy or transoral sialolithotomy with sialodochoplasty [11]. The surgical management of deeply situated submandibular gland and intraglandular segment stones is complex, challenging, and requires clinical expertise [11]. Eighty-five percent of sialoliths occur in the Wharton's duct, and only 15% occur within the parenchyma of the submandibular gland [12]. Ultrasonography has a higher diagnostic accuracy for identifying sialolith in the parotid gland than the submandibular gland [13]. Cone-beam computed tomography (CBCT) has recently helped visualize complex sialolithiasis [14]. Magnetic resonance sialography with three-dimensional extended-phase conjugate-symmetry rapid spin-echo sequence (3D EXPRESS) MRI enables reliable prediction of salivary gland calculi but not for salivary stones of less than 1-2 mm in diameter, which is often confused with inspissated mucus [15]. The treatment of submandibular sialolith depends on the size and anatomical location of the calculi [16]. Thong et al. reported an asymptomatic painless giant-sized submandibular sialolith of about 26 mm diameter [17]. Patients with submandibular sialoliths present clinically with acute onset of swelling in the neck with fever [18]. More extraparenchymally located submandibular salivary stones are managed by surgical excision of the affected submandibular gland by the Risdon approach [18]. Small, easily accessible submandibular salivary calculi are managed with conservative methods, such as bimanual palpation or milking of the salivary gland and palliative care. In contrast, more extensive, difficult-to-reach salivary stones need surgical removal of the affected salivary gland [19].

Clinical significance

This case report describes a simple manual chairside technique for retrieving a superficially located submandibular sialolith near the sublingual caruncle, where the orifice of the submandibular duct opens. The method involves a gentle massage of the submandibular salivary gland through bidigital palpation or milking of the gland. Importantly, this technique eliminates the need for complex surgical procedures for superficially located submandibular sialolith proximal to sublingual caruncle, thereby reducing potential risks and complications. 

## Conclusions

A proper history taking and clinical examination aid in diagnosing submandibular sialadenitis caused secondary to a sialolith. The treatment of a sialolith is contingent on the size and exact location of the sialolith in the submandibular salivary gland duct. Sialoliths, which are superficially located near the hilum at a more superficial location, can be easily retrieved by gently massaging the submandibular gland. However, those located deeper inside the parenchyma necessitate the removal of the salivary gland. The use of proper imaging techniques, coupled with clinical expertise, is crucial in finding and retrieving a submandibular sialolith. In cases of salivary gland enlargement, the clinician must also screen for the possibility of sialolithiasis. Early diagnosis and prompt treatment of sialolithiasis are crucial to prevent damage to the salivary glands and improve the quality of life.
